# 2252: Decreasing loss to follow up after surgery for meningioma

**DOI:** 10.1017/cts.2017.255

**Published:** 2018-05-10

**Authors:** Whitney Muhlestein, Alice Song, David G. Schlundt, Lola B. Chambless

## Abstract

OBJECTIVES/SPECIFIC AIMS: “Loss to follow up” is a common phenomenon and challenge in clinical medicine. Missed appointments are a well-documented source of waste in the health care system, and can lead to strained patient-physician relationships and inferior quality of care. Meningiomas are relatively common, benign tumors that arise from the dural coverings of the brain. Although complete surgical resection is considered curative, surgically excised meningiomas have a well-documented propensity to recur, necessitating continued imaging surveillance of postresection patients. A recent retrospective study at our institute demonstrated that 20% of postresection patients fail to return for follow up within a year of their surgery. Although social determinants of health have been associated with failure to follow up in this population, there has been no research identifying patient-reported barriers that result in loss to follow up in this patient population. The purpose of this study is to identify specific barriers that prevent patients from returning for surveillance. METHODS/STUDY POPULATION: We used an IRB approved, prospective brain tumor clinical database to identify patients who underwent surgical resection of intracranial meningioma at our institution between 2001 and 2013. “Loss to follow up” was defined as failure to attend follow-up appointments with neurosurgery, radiation oncology, or neuro-oncology within a year of the most recent assigned follow-up interval, as recorded in the electronic medical record. Structured interviews were conducted with patients who met study criteria and specific barriers to follow-up were elicited, transcribed, and coded. In 2 cases, a primary caregiver participated in all or portions of the interview with the patient. A general assessment of patient knowledge about meningioma and a screening for basic health literacy were also conducted. RESULTS/ANTICIPATED RESULTS: There were 80 patients in the brain tumor clinical database met chart review criteria for inclusion in the study. A total of 9 structured interviews were conducted; 1 interview was excluded from analysis for failure to meet study criteria. In total, 24 unique obstacles to follow up were recorded. These were stratified and grouped into 4 broad categories: 2 of 8 (25%) patients identified environmental factors, including distance to appointment and challenges with insurance coverage as barriers to follow up; 2 patients (25%) identified psychosocial factors, including poor communication with and distrust of their neurosurgeon as barriers to follow up; 2 (25%) patients identified health factors, including poor health and old age, as barriers to follow up; 6 patients identified healthcare systems factors as barriers to follow up, with 6 patients (75%) reporting seeing a non-specialist for follow up after surgery and 4 patients (50%) reporting not being told by their neurosurgeon that they would need continued follow up. Of those patients seen by non-specialists, only 1 reported any recent brain imaging by those providers. All patients had limited to no prior knowledge of meningiomas before their diagnosis. Four (50%) patients reported satisfaction with the level education about meningiomas they received from their physician. Of these patients, 3 (75%) correctly reported that meningiomas may recur following surgery. Of the patients who did not report satisfaction with physician counseling, 3 (75%) did not realize that meningiomas can recur. DISCUSSION/SIGNIFICANCE OF IMPACT: Healthcare system factors, including uncoordinated transition of postoperative care to non-neurosurgeons and uncertain postoperative surveillance schedules, represent that most common patient-identified barriers to follow up after meningioma resection. Improving transition of care from specialists to non-specialists, including designation of appropriate imaging surveillance schedules, as well as improving communication between specialists and patients about the need for continued follow up, represent clear points for intervention that could improve care for this patient population. In addition, consistent and clear counseling about meningioma and its disease course may reduce loss to follow up following meningioma resection. It is important to note, however, that the small sample size represents a significant limitation of the study.

